# Differentiating fulminant EBV infection complicated by HLH from Lymphoma: report of a case and a brief literature review

**DOI:** 10.1186/s13000-023-01307-x

**Published:** 2023-02-22

**Authors:** Bradley Zehr, Kristina Brannock, Rebecca Wyma, Samir B. Kahwash

**Affiliations:** 1 Department of Pathology, College of Medicine, The Ohio State University, Columbus, OH USA; 2 Department of Pathology and Laboratory Medicine, Nationwide Children’s Hospital, 700 Children’s Drive, Columbus, Ohio 43205-2644 USA; 3 College of Medicine, The Ohio State University, Columbus, OH USA

**Keywords:** Epstein-Barr virus (EBV), Hemophagocytic lymphohistiocytosis (HLH), Classic Hodgkin lymphoma (cHL)

## Abstract

Epstein-Barr virus (EBV) infection may present with fulminant constitutional symptoms, cytopenia(s), and systemic lymphadenopathy, raising clinical suspicion for lymphoma and prompting lymph node and bone marrow biopsies. At the microscopic level, the histopathologic findings in cases of acute EBV lymphadenitis may mimic certain lymphoid neoplasms, creating a range of differential diagnoses and diagnostic pitfalls.

We present a case of fulminant EBV infection in an adolescent whose clinical and radiographic findings led to lymph node and bone marrow biopsies to rule out lymphoma. One week after being diagnosed with acute EBV infection (infectious mononucleosis), a 17-year-old Caucasian male presented with worsening symptoms including persistent fever, progressive, painful lymphadenopathy, and splenomegaly. A peripheral blood smear showed lymphocytosis with many reactive lymphocytes, anemia, and thrombocytopenia. Laboratory studies showed elevated ferritin, triglycerides, and soluble IL-2/CD25. A cervical lymph node biopsy demonstrated an EBV-positive, reactive B-immunoblast proliferation with large atypical lymphoid cells mimicking Reed-Sternberg cells of Hodgkin lymphoma, in addition to patchy vasculitis, coagulative necrosis, and prominent hemophagocytic activity. Bilateral bone marrow biopsies showed a hypercellular marrow with patchy infiltrates of similar EBV-positive, large atypical lymphoid cells, as well as prominent hemophagocytic activity. The diagnosis of acute EBV associated lymphoproliferation with concurrent hemophagocytic lymphohistiocytosis (HLH) was rendered.

Recognition of common and uncommon clinical presentations of acute EBV infection is essential, particularly when histopathologic findings raise suspicion for a possible hematolymphoid neoplasm. Both the lymph node architectural and viral cytopathic changes observed in EBV lymphadenitis exhibit significant morphologic overlap with classic Hodgkin lymphoma (cHL) and several other lymphomas, including anaplastic large cell lymphoma, diffuse large B cell lymphoma, and angioimmunoblastic T cell lymphoma. Recognition of immunohistochemical staining patterns in EBV lymphadenitis is critical to avoid misdiagnosis. Conversely, bona fide lymphoma, particularly cHL, can masquerade as EBV infection. We provide a concise discussion and tables of the histopathologic differential diagnosis of EBV lymphadenitis, including cHL and other lymphomas. Pathologists should include acute EBV infection within the differential diagnosis when confronted with clinical and pathologic findings concerning for lymphoma, particularly in adolescents and young adults.

**Supplementary Information:**

The online version contains supplementary material available at 10.1186/s13000-023-01307-x.

## Introduction

Epstein-Barr virus (EBV) infection may present or evolve during the acute phase to a fulminant disease manifesting with severe constitutional symptoms, systemic lymphadenopathy, and associated cytopenia(s). In such clinical scenarios, tissue biopsy may be performed to exclude an underlying lymphoproliferative disorder. A lymph node and/or bone marrow biopsy in the setting of active EBV infection can create a challenging diagnostic dilemma as the histopathologic findings may closely mimic certain lymphomas, raising the possibility of diagnostic pitfalls. The astute pathologist must be able to separate the morphologically worrisome changes associated with acute EBV infection (infectious mononucleosis) from those of the many EBV-associated lymphoproliferative disorders included in the World Health Organization (WHO) Classification of Hematolymphoid Tumors, 5^th^edition, online beta version (Supplement) [1]. The goal of this brief report is to highlight the diagnostic challenges of EBV lymphadenitis by presenting a case of fulminant EBV infection in an adolescent male whose striking clinical and radiographic findings led to lymph node and bone marrow biopsies in order to rule out lymphoma.

## Case report

### Clinical history

A previously healthy 17-year-old Caucasian male who had recently been diagnosed with acute EBV infection (after presenting with a febrile illness and found to have positive EBV IgM serology and serum viral load= 958,940 copies/mL by PCR) re-presented seven days post initial diagnosis with worsening symptoms including persistent fever and progressive, painful cervical lymphadenopathy. His serum EBV viral load had increased to 1,261,700 copies/mL, and he was found to have leukocytosis (WBC 16,400/uL) with lymphocytosis (lymphocytes 9800/uL), anemia (hemoglobin 9.6 g/dL), and worsening thrombocytopenia (platelet count 121,000/uL). CT scans of the neck and chest showed marked bilateral cervical lymphadenopathy extending to the bilateral supraclavicular regions, mediastinum, perihilar region of the thorax, and bilateral axillae. A CT scan of the abdomen and pelvis revealed multifocal lymphadenopathy and marked splenomegaly, raising clinical and radiologic suspicion for a possible lymphoproliferative disorder. A right cervical lymph node excisional biopsy and bilateral iliac crest bone marrow biopsies were performed.

### Pathologic findings

**Fig. 1:**
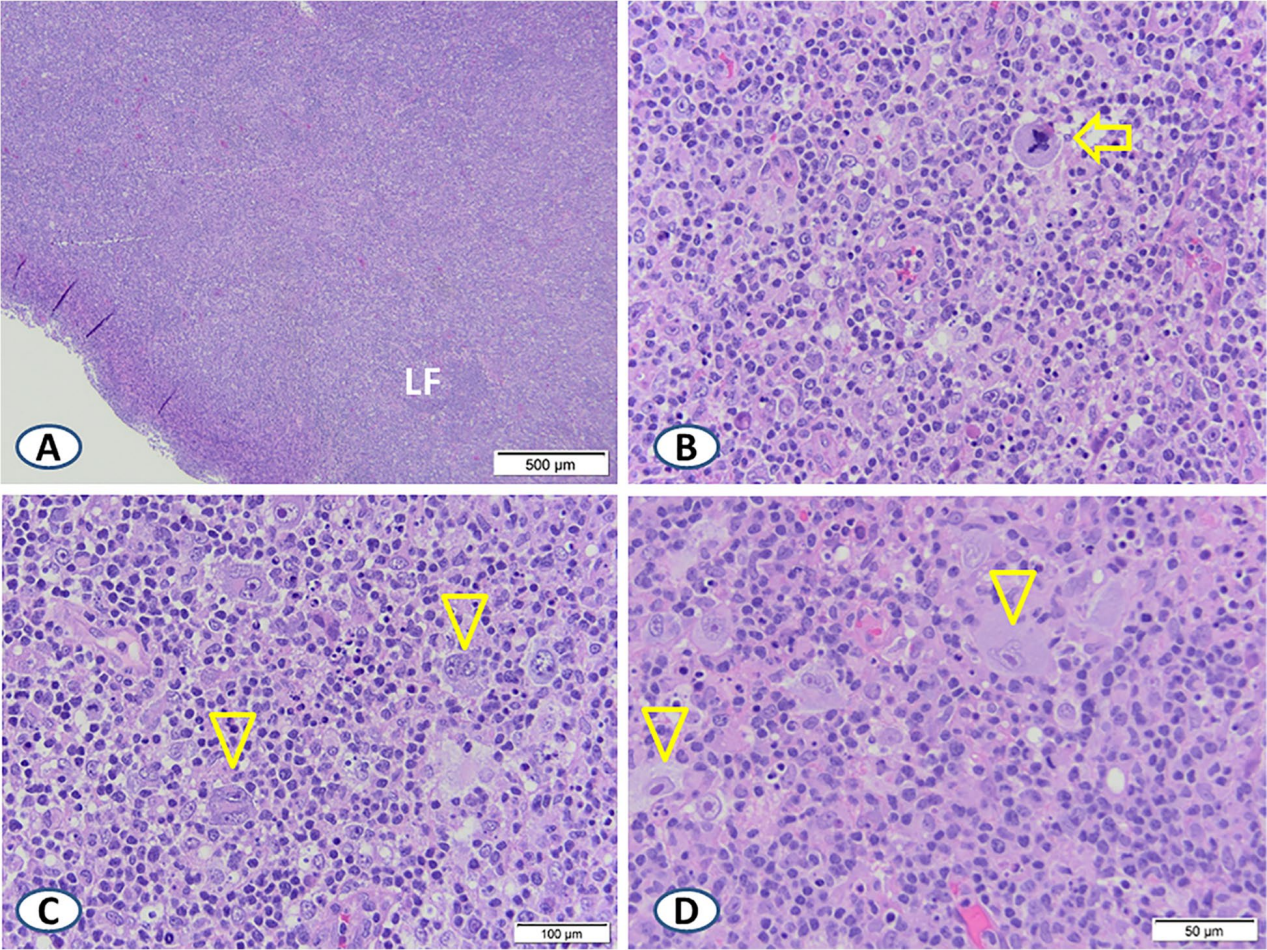
Selected H&E sections of the cervical lymph node excisional biopsy. Note the mottled architecture with subtle evidence of a residual lymphoid follicle (LF) in **A**, and a mixed population of small, medium, and large-sized lymphoid cells including mitoses (arrow) in **B**. Areas rich in large lymphoid cells with lobulated nuclei, prominent nucleoli, and multinucleation are shown in **C** (arrowheads). Areas where large cells show viral-type nucleoli (arrowheads) are demonstrated in **D**

The cervical lymph node excisional biopsy showed pieces of lymphoid tissue with mottled and effaced architecture replaced by sheets of variably sized lymphoid cells admixed with areas of increased phagocytic macrophages. The lymphoid cells were predominantly small to intermediate sized, with areas of admixed large, reactive immunoblasts along with cells exhibiting prominent nucleoli, multinucleation, and viral-type nuclear inclusions (Fig. 1A, B, C, and D). Plasma cells were focally increased with no significant number of eosinophils present.

**Fig. 2:**
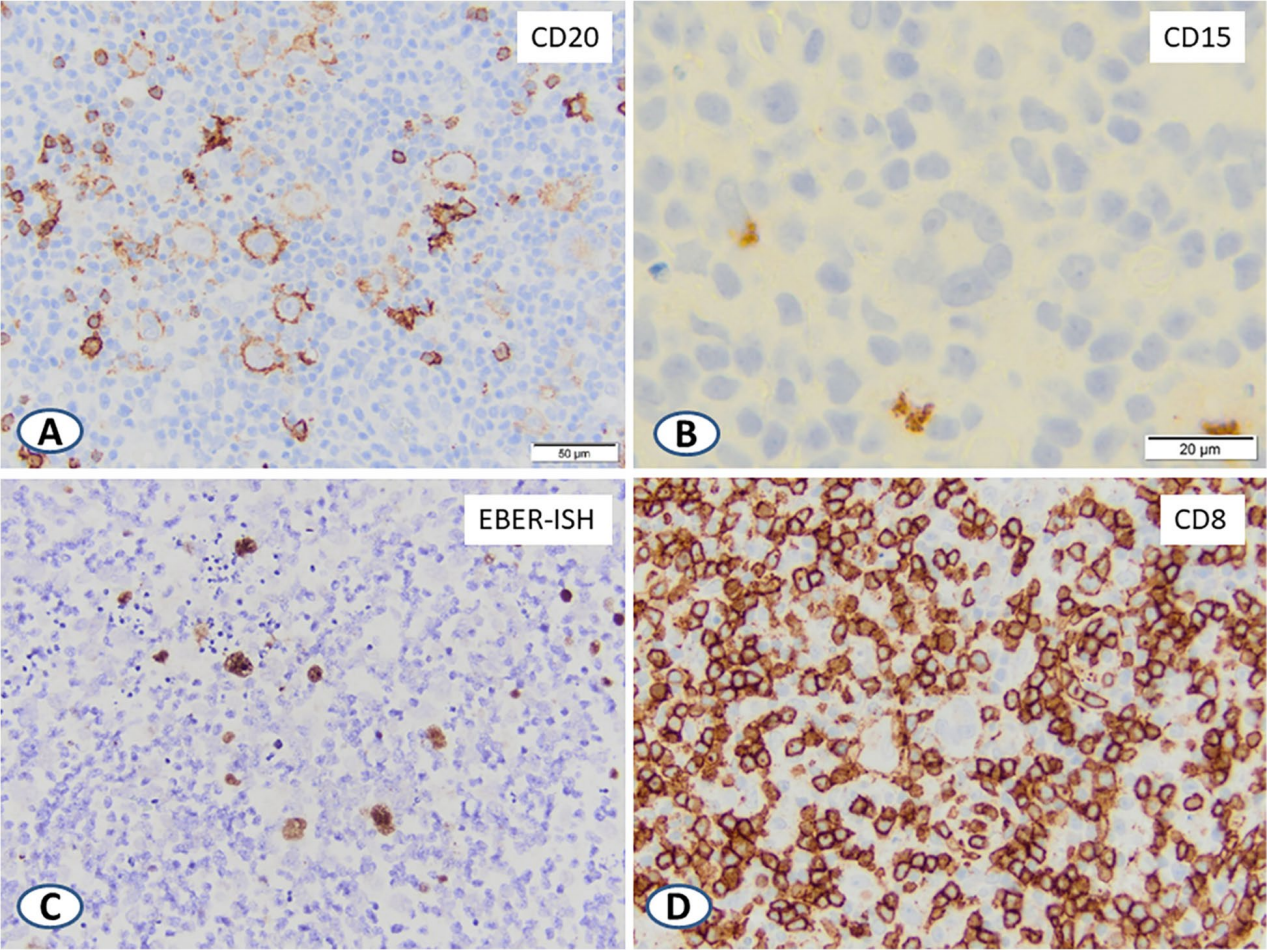
Selected IHC stains of the cervical lymph node excisional biopsy showing large cells expressing CD20 (**A**) and negative CD15 (**B**). Encoded EBV RNA (**C**) positivity is seen mostly in larger cells, and CD8 (**D**) positive T- cells predominated background small lymphocytes

Immunohistochemical (IHC) stains showed the large lymphoid cells to be positive for CD20, PAX-5 (bright), CD30, and EBV-encoded RNA in situ hybridization (EBER-ISH), consistent with activated, EBV-infected B-cells. These cells were negative for CD15 and ALK-1. CD3 highlighted background T-cells with a predominance of CD8-positive over CD4-positive T-cells (Fig. 2A, B, C, and D). EBER-ISH was negative in the background T-cells.

**Fig. 3:**
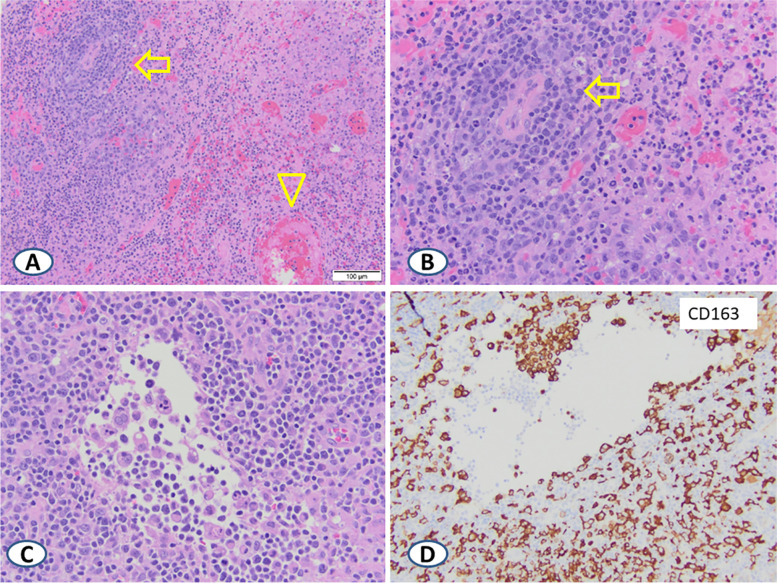
Areas of vasculitis (**A** and **B**, arrows) and thrombosed vessels resulting in coagulative necrosis (A, arrowhead). There was a marked increase in hemophagocytic activity, especially within vascular spaces (**C**) with CD163 highlighting macrophages (**D**)

Patchy areas of vasculitis with associated coagulative necrosis were noted, with inflammatory cells of variable sizes infiltrating and surrounding vessels (Fig. 3A and B). Phagocytic macrophages were prominent in some vascular spaces (Fig. 3C). CD163 highlighted an extensive network of large, activated macrophages (Fig. 3D). EMA was negative in the large lymphoid cells.

A portion of the lymph node tissue was sent for flow cytometry, which demonstrated an inverted CD4:CD8 ratio but no evidence of a clonal T-cell or B-cell population.

A review of peripheral blood smear morphology showed lymphocytosis with reactive atypical lymphocytes, mildly left-shifted granulocytes with toxic granulation, mild normocytic anemia, and mild thrombocytopenia.

**Fig. 4:**
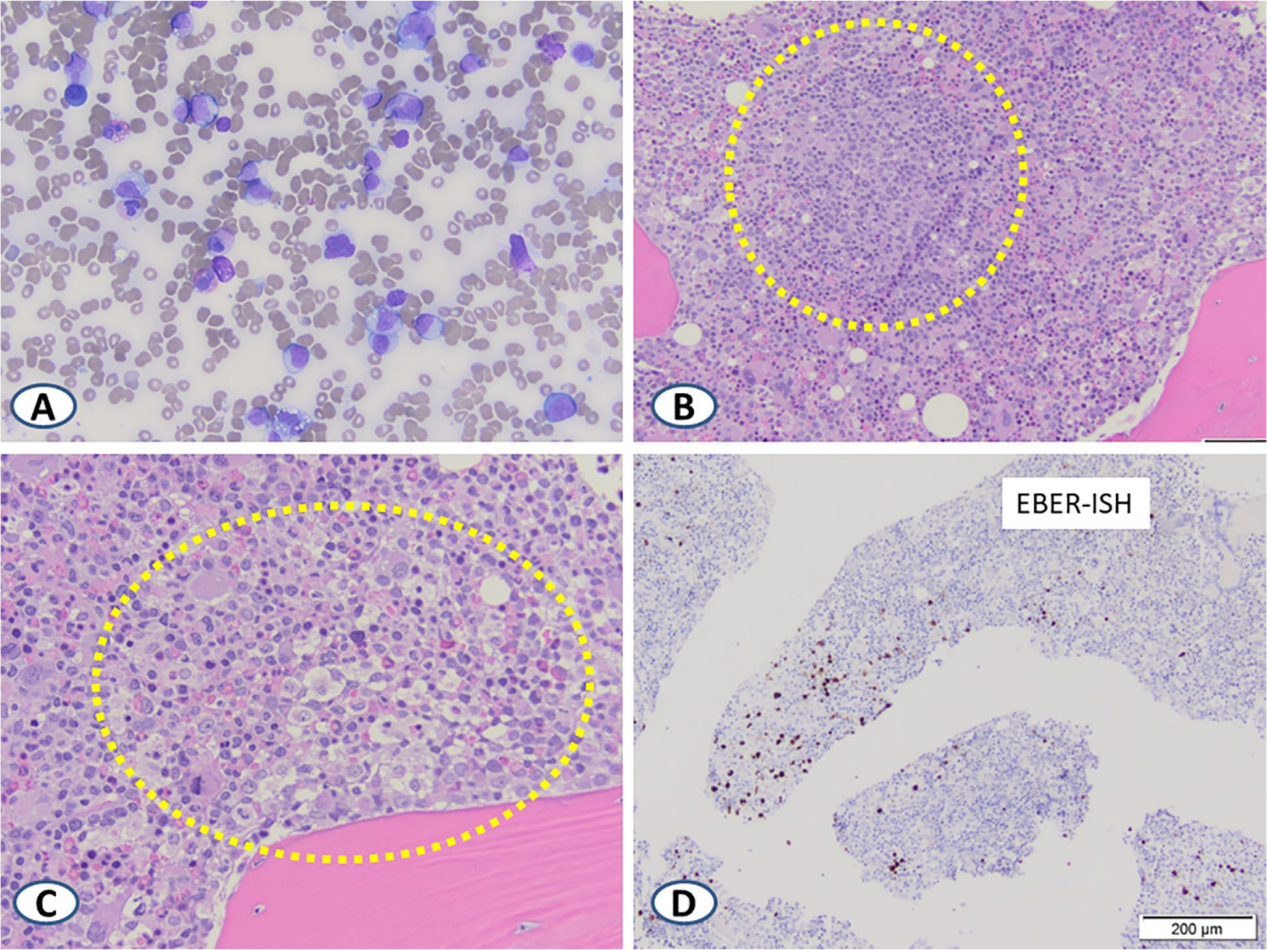
Wright-Giemsa-stained bone marrow aspirate smear showing increased reactive atypical lymphocytes and monocytes (**A**) and increased phagocytic macrophages (**B** and **C**, encircled). As noted in lymph node, EBER positivity – shown here on section from bone marrow biopsy – decorated larger reactive lymphoid cells (**D**)

Bilateral bone marrow core biopsies and aspirates showed hypercellular marrow (80-90%) with patchy infiltrates of atypical lymphocytes including large lymphoid cells featuring large nuclei and viral-type inclusions (Fig. 4A). Frequent hemophagocytic macrophages were also present (Fig. 4B and C). Immunohistochemical staining demonstrated that the increased lymphocytes were predominantly CD3-positive T-cells, with a predominance of CD8-positive over CD4-positive T-cells. The larger lymphoid cells were highlighted by IHC staining for CD20 and EBER-ISH, consistent with reactive, EBV-infected B-cells (Fig. 4D). CD163 stain highlighted the prominent phagocytic macrophages.

Based on these morphologic and immunophenotypic findings, the diagnosis of acute EBV associated lymphoproliferation with suspected concurrent hemophagocytic lymphohistiocytosis (HLH) was rendered. Testing for T-cell receptor (TCR) gene rearrangement was initiated to further exclude an EBV-driven T-cell lymphoproliferative disorder.

Additional laboratory workup for HLH was notable for an elevated ferritin (1,684 ng/mL, reference range 31-294 ng/mL), elevated triglycerides (386 mg/dL, reference range 29-200 mg/dL), normal fibrinogen level (309 mg/dL, reference range 170-410 mg/dL), normal NK cell frequency and absolute count, and an elevated soluble IL-2R/CD25 (19,338 U/mL, reference range 137-838 U/mL). These findings, in conjunction with the fever, splenomegaly, and histologic evidence of hemophagocytosis, met the HLH-2004 criteria for a diagnosis of HLH [2].

### Laboratory and clinical follow-up

TCR gamma/beta (TCR-G/B) clonality studies, performed on the lymph node biopsy to further exclude an EBV-driven T-cell lymphoproliferative disorder, showed polyclonal TCRG and skewed/oligoclonal TCRB but no definitive clonal T-cell population.

A subsequent next-generation sequencing (NGS) panel performed on peripheral blood for common genetic variants associated with familial HLH was negative. Cytogenetic studies on the bone marrow aspirate revealed an inverted chromosome 6, likely representing a constitutional abnormality unrelated to the histopathologic findings.

The patient was treated with high-dose dexamethasone for virus-associated HLH, leading to rapid, marked improvement in lymphadenopathy, splenomegaly, and symptoms. Within ten days of steroid initiation, he had recovered to his baseline state except for persistent mild fatigue, and the EBV viral load had markedly decreased to 24,784 copies/mL. To our knowledge, the patient remained healthy at ten months post-diagnosis.

## Discussion

We present a case of fulminant acute EBV infection complicated by HLH in a 17-year-old Caucasian male whose clinical presentation raised the possibility of lymphoproliferative disorder. Although tissue biopsy is usually unnecessary in cases of acute EBV infection, at times the constellation of clinical, laboratory, and imaging findings necessitate a lymph node and/or bone marrow biopsy to evaluate for a possible lymphoid neoplasm. Caution is warranted when evaluating tissue biopsies in the setting of acute EBV infection, as the pathologic findings can often mimic lymphoma and potentially result in misdiagnosis.

**Fig. 5:**
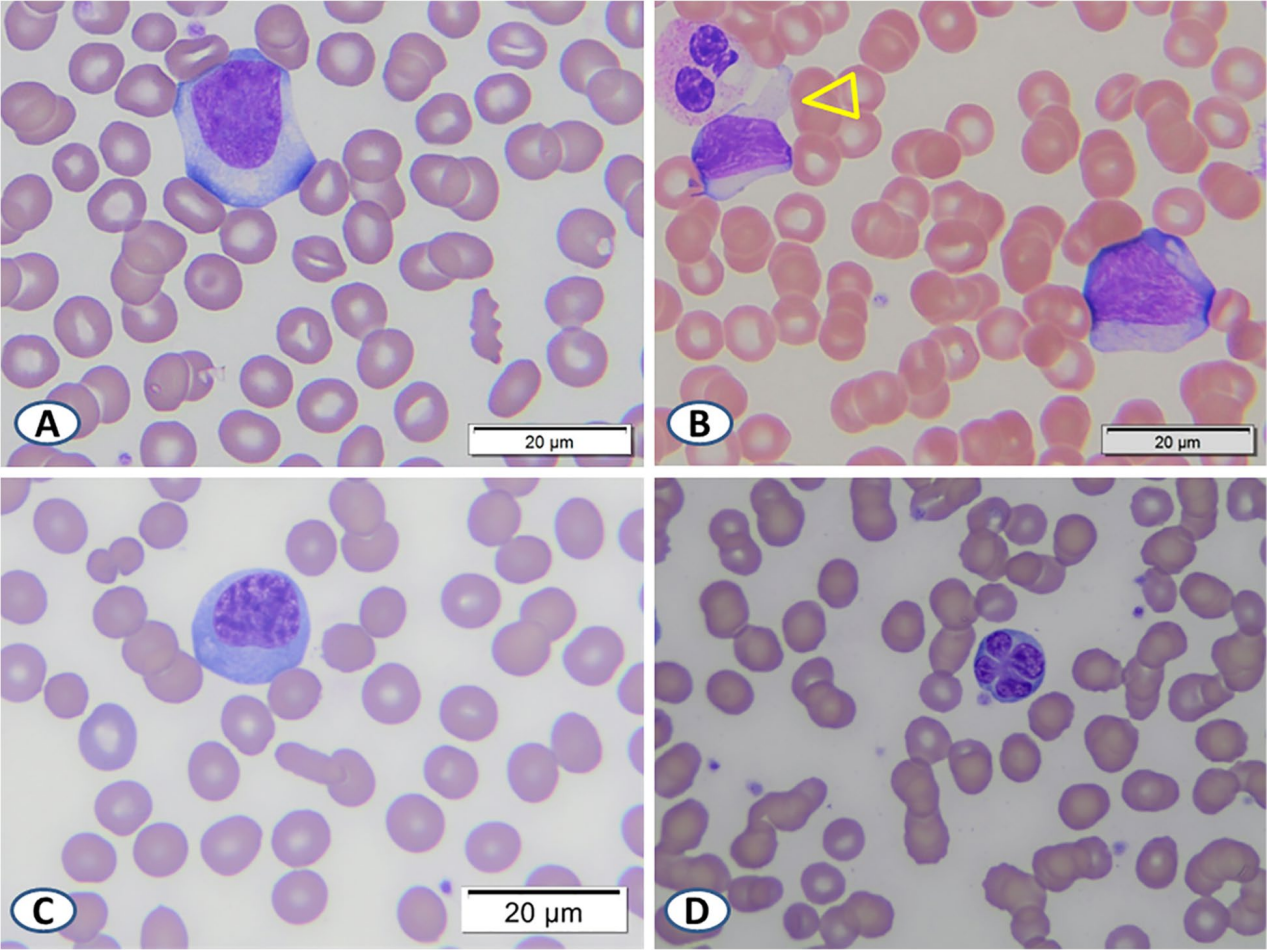
Representative images of typical reactive lymphocytes in the peripheral blood smear in acute EBV infection. Type 2 Downey cells – large, atypical lymphocytes with moderate to abundant scalloped cytoplasm – are the most common (**A** and **B**) with some showing pseudopod-like cytoplasmic extensions (B, arrowhead). Plasmacytoid lymphocytes (**C**) and those with clover-leaf nuclei (**D**) are less commonly encountered. (Note: images in this figure were taken from confirmed archival cases.)

Recognition of common and uncommon clinical presentations of acute EBV infection is essential, particularly when histopathologic findings raise suspicion for a possible hematolymphoid neoplasm. The common signs and symptoms of acute EBV infection include the classic triad of fever, pharyngitis, and lymphadenopathy. Malaise, headache, and splenomegaly occur in about half of cases and anorexia in a subset of cases [3]. Infectious mononucleosis most commonly presents in adolescents and young adults but may occur at any age. Leukocytosis with lymphocytosis and thrombocytopenia are common, but patients, especially the young, may also occasionally present with bi-cytopenia or even pancytopenia. A peripheral blood smear in cases of infectious mononucleosis typically shows a transient lymphocytosis composed of enlarged, reactive lymphocytes with abundant, pale to deeply basophilic cytoplasm and scalloped cell borders, often molding to adjacent erythrocytes, also known as type 2 Downey cells [4] (Fig. 5A and B).

Several distinct EBV-related viral syndromes occur and can usually be distinguished based on the clinical presentation and whether the infected lymphocytes are B-cells or T-cells [5, 6]. Acute EBV infection (infectious mononucleosis) and X-linked lymphoproliferative disease involve EBV-infected CD21-positive B-cells, with the former typically exhibiting a mild, self-limited clinical course and the latter presenting with more severe illness and molecular evidence of a mutated SH2D1A gene [5]. Systemic EBV-positive T-cell lymphoma of childhood, an umbrella term which encompasses several overlapping entities, involve EBV-infected CD8-positive T-cells, often with evidence of T-cell clonality and associated HLH [7]. Chronic active EBV infection (CAEBV) may involve EBV-infected B-cells, T-cells, or NK cells and is characterized by persistent/recurrent EBV infection spanning at least 3 months but without definitive evidence of a clonal lymphoid population [7].

EBV-positive lymphoproliferative disorders occur worldwide. Systemic EBV-positive T-cell lymphoma of childhood and CAEBV are most prevalent in East Asia but have also been reported in Central and South America and rarely in Western countries. CAEBV has been seen rarely in African countries. The mixed cellularity and lymphocyte-depleted subtypes of cHL are the subtypes that are most frequently positive for EBV [1], and these subtypes are more frequently seen in the developing world, particularly within the HIV-positive population.

**Fig. 6:**
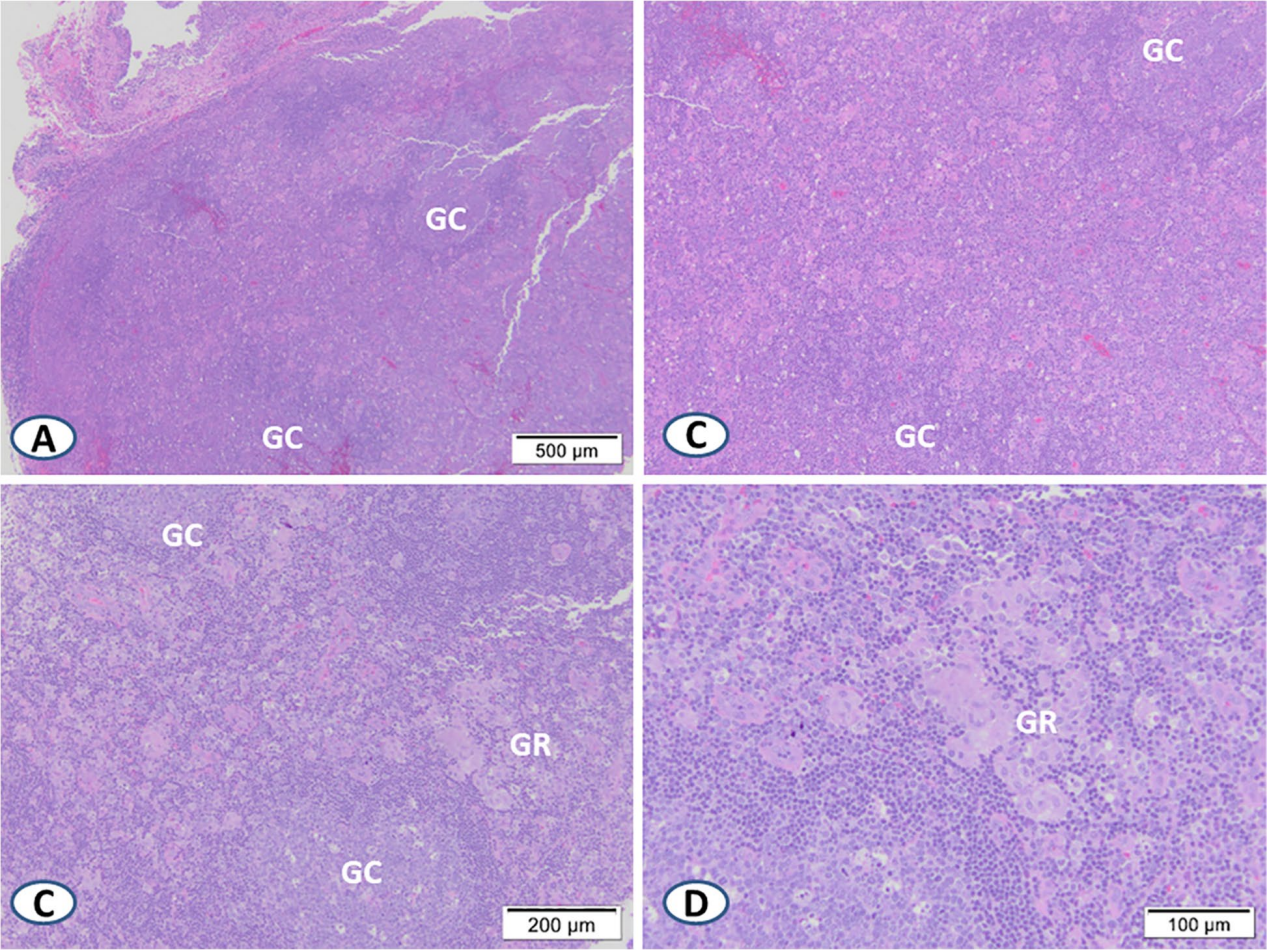
Selected H&E sections of typical lymph node architecture in acute EBV infection showing expanded interfollicular zones separated by remnants of lymphoid follicles with germinal centers (GC) (**A** and **B**). Sheets of epithelioid histiocytes forming ill-defined “soft” granulomas (GR) can be seen (**C** and **D**). (Note: images in this figure were taken from confirmed archival cases.)

Both the lymph node architectural changes and viral cytopathic changes observed in EBV lymphadenitis exhibit significant morphologic overlap with cHL and several other lymphomas (Tables 1 and 2). EBV lymphadenitis and cHL often exhibit distorted lymph node architecture with a mixed infiltrate composed of lymphocytes, plasma cells, and histiocytes with prominent, enlarged CD30-positive lymphoid cells, often with Reed-Sternberg-like morphology. On tissue sections, EBV-infected B-cells exhibit characteristic cytopathologic changes including cellular and nuclear enlargement, possible multinucleation, and prominent basophilic nucleoli, consistent with immunoblast morphology and potentially mimicking Reed-Sternberg cells or variants of cHL [8]. When present, a mottled appearance with remnants of reactive follicles separated by expanded inter-follicular zones favors EBV lymphadenitis (Fig. 6A and B).

**Table 1:** Comparison of low- and medium-power microscopic findings in EBV lymphadenitis versus selected lymphomas

	**Active EBV Lymphadenitis**	**cHL**	**ALCL**	**DLBCL Non-GCB Subtype**	**AITL**
Lymph Node Architecture	Mottled, partially preserved	Usually Not preserved	Usually Not preserved (Except in early involvement)	Not preserved	Not preserved, but sparing of peripheral cortical sinuses
Cell Size Dichotomy Between Large Atypical & Background Cells (versus a spectrum of variably sized cells)^a^	No	Yes	No	No	No
Necrosis	Yes, common, usually patchy	Yes (in nodular sclerosis subtype)	Not common	Yes	Rare
Vasculitis & Vessel Wall Infiltrates	Common	Uncommon	Uncommon	Uncommon	Uncommon
Granulomas	Rare, ill-defined “soft” granulomas	Rare, “soft” granuloma (mostly in mixed cellularity subtype)	No	No	No, but may have increased reactive histiocytes

^a^Notable exceptions include the rare small cell variant of ALCL and EBV positive DLBCL

**Table 2:** Comparison of high-power microscopic findings and ancillary testing in EBV lymphadenitis versus selected lymphomas

	**Active EBV Lymphadenitis**	**cHL**	**ALCL**	**DLBCL Non-GCB Subtype**	**AITL**
**Large Atypical Cells**	Viral cytopathic effects, polymorphic lymphoid cells, immunoblasts, R-S-like cells	R-S cells	Small, medium, and large anaplastic cells, hallmark cells, occasional doughnut cells, R-S-like cells	Large atypical lymphoid cells, immunoblasts, centroblasts, R-S-like cells	Small to intermediate cells with clear cytoplasm and minimal atypia, immunoblasts, R-S-like cells
	Positive: CD30, CD20, MUM1, OCT-2, BOB.1	Positive: CD30, ± CD15, PAX5 (weak), -/ + CD20, MUM1	Positive: CD30, ± ALK-1, EMA, CD2, CD3, CD4, and/or CD5	Positive: CD30, CD20, MUM1	Reactive Immunoblasts: Positive for CD30, CD20
	Negative: CD15	Negative: CD45, OCT2, BOB.1	Negative: CD15	Negative: CD10, BCL6	Negative: CD15
**Small Background Cells**	T-cells (CD8 + predominant); No significant eosinophils	T-cells (CD4 + predominant) Eosinophils, plasma cells, histiocytes	Lymphocytes, histiocytes; No eosinophils	Lymphocytes, plasma cells, histiocytes	Eosinophils, lymphocytes, plasma cells, histiocytes
**EBER-ISH**	Positive (large cells and small cells)	± Positive (+ mostly in large cells)	Negative (always)	± Positive (often + in immune deficiency related subtypes)	Positive in background B-immunoblasts, negative in neoplastic T-cells
**EBV Serology (IgM)**	Positive	Negative	Negative	Negative	Negative
**EBV PCR (peripheral blood)**	Positive (high viral load)	Positive (low viral load) or Negative	Negative	Positive/ Negative	Positive/ Negative

Prominent eosinophil infiltrates are characteristic of cHL, although not seen in every case, particularly the lymphocyte-rich subtype. In contrast, EBV lymphadenitis shows T-cell predominance in the background lymphocytes, with a preponderance of CD8-positive T-cells and typically no eosinophils. cHL usually exhibits a distinct dichotomy between the prominent large neoplastic cells and the small background reactive cells, while acute EBV infection proliferation features a spectrum from small to medium or large sized cells. Venous proliferation with high endothelial venules is common in EBV lymphadenitis but generally not found in cHL. Focal or patchy necrosis is common in acute EBV lymphadenitis while uncommon in cHL, except for the nodular sclerosis subtype, where microscopic necrosis can be seen surrounded by a rim of R-S variant cells. Collections of epithelioid histiocytes forming “soft” granulomas may infrequently be seen in cHL (usually mixed cellularity subtype) but may also be seen in EBV lymphadenitis (Fig. 6C and D).

Recognition of immunohistochemical staining patterns in the differential of EBV lymphadenitis is critical to avoid misdiagnosis. Reed-Sternberg (R-S) cells of cHL typically stain positive for CD30, CD15, PAX5 (weak), and MUM1 but negative for CD20 and CD45. The immunoblasts and R-S-like cells of EBV lymphadenitis are typically positive for CD20, CD30, MUM1, OCT-2, and BOB-1 but negative for CD15 and should be EBER-ISH positive. Occasionally, cHL R-S cells may be CD15 negative, CD20 positive, and/or EBER-ISH positive, leading to potential diagnostic uncertainty. R-S cells typically show decreased or lost expression of OCT-2 or BOB-1, whereas reactive immunoblasts typically (but not always) retain OCT-2 and BOB-1 expression, which could potentially assist in distinguishing cHL from EBV lymphadenitis [9–12].

Misdiagnosis of EBV infection as diffuse large B-cell lymphoma (DLBCL) has also been reported, as the CD20 + /CD30 + /MUM1 + /EBER-ISH + immunoblasts of EBV infection may be present in sheets with associated necrosis and marked distortion of node architecture [9]. In such cases, the immunoblasts exhibited non-germinal center immunophenotype (CD10-/BCL6-/MUM1 +), and therefore the possibility of DLBCL with non-germinal center phenotype could not be excluded. Additionally, T-cell/histiocyte-rich large B-cell lymphoma may also mimic acute EBV infection, as both entities feature scattered large, atypical B-cells amid a dense background of T-cells and histiocytes. The diagnostic distinction may require clinical, radiographic, serologic, and cytogenetic correlation and molecular testing for clonal gene rearrangement. Demonstration of kappa or lambda light chain restriction in the large cells supports a DLBCL diagnosis, whereas the immunoblasts of EBV lymphadenitis are expected to be polytypic for kappa and lambda. DLBCL with germinal center phenotype is distinguishable by its positivity for CD10 and/or BCL6 and negativity for MUM1, an immunophenotype distinct from immunoblasts of EBV lymphadenitis.

Flow cytometry may be useful for distinguishing EBV lymphadenitis from other non-Hodgkin, mature B-cell lymphomas, in addition to DLBCL. Given that immunohistochemistry and in situ hybridization for kappa and lambda light chains are frequently non-diagnostic in tissue biopsies, flow cytometry is generally the preferred methodology for assessing B-cell clonality in non-Hodgkin B-cell lymphomas, which typically demonstrate monoclonality of the neoplastic B-cells with surface kappa and lambda light chains, whereas EBV lymphadenitis should demonstrate a polyclonal B-cell population.

EBV lymphadenitis may also potentially mimic certain T-cell lymphomas, particularly angioimmunoblastic T-cell lymphoma (AITL) and anaplastic large cell lymphoma (ALCL). Steciuk et al. report a case of marked EBV viremia in an immunosuppressed adolescent male who underwent inguinal lymph node excisional biopsy [13]. Histologic sections showed features highly suggestive of AITL, including distorted node architecture with clusters and sheets of monotonous clear cells with admixed arborizing endothelial venules lined by plump endothelial cells, regressed “moth-eaten” germinal centers, and extensive fibrosis. Areas with abundant immunoblasts were also present, and IHC staining showed the immunoblasts were positive for CD20, CD30, and EBER-ISH but negative for CD15. The monotonous clear cells were positive for CD3, CD4, and CD5, weakly positive for PD-1, and negative for CD10. TCR-gamma clonality studies demonstrated a clonal T-cell population. However, a diagnosis of AITL was not rendered, and instead the findings were favored to represent EBV infection. The patient’s lymphadenopathy and EBV viremia resolved with discontinuation of immunosuppressive therapy, leading the authors to conclude that the patient had a benign, immunosuppression-related lymphoproliferative disorder.

ALCL, like cHL and EBV lymphadenitis, features large, atypical CD30-positive lymphoid cells that may occasionally show Reed-Sternberg-like morphology. The so-called hallmark cells, which feature horseshoe-shaped or reniform nuclei, are a distinguishing histologic feature of ALCL [14]. EMA positivity in the large cells favors ALCL, which may be ALK-1 positive or ALK-1 negative but is usually positive for at least one T-cell marker such as CD2, CD3, CD4, or CD5. EBER-ISH positivity of the large cells essentially excludes a diagnosis of ALCL but is seen in a significant subset of cHL cases, particularly the mixed cellularity and lymphocyte-depleted subtypes [1].

Hepatosplenic T-cell lymphoma (HSTCL) is an additional rare potential mimicker of EBV lymphadenitis. Both often present with constitutional symptoms, splenomegaly, and cytopenias in adolescents and young adults. Both feature a T-cell population with abnormal immunophenotype by flow cytometry, and both may exhibit associated HLH. However, HSTCL is not an EBV-associated neoplasm, and typically HSTCL does not present with generalized or cervical lymphadenopathy but rather localized perisplenic lymphadenopathy. Additionally, the neoplastic T-cells of HSTCL are typically dual CD4-/CD8-, whereas the reactive T-cells associated with acute EBV infection are CD8 + [15].

Flow cytometry may aid in separating EBV lymphadenitis from T-cell lymphomas such as AITL and ALCL. If T-cell abnormalities are detected by flow cytometry, such as loss of pan T-cell antigens or a skewed CD4:CD8 ratio, these may suggest a T-cell lymphoma. However, caution is warranted as typically flow cytometric analysis will detect a CD8 + T-cell population with bright HLA-DR + and CD38 + , down-regulated CD7, and possibly down-regulated CD5 in acute infectious mononucleosis [16].

Similarly, monoclonality by TCR gene rearrangement studies may increase the index of suspicion for T-cell lymphoma. Importantly, not all T-cell lymphomas will necessarily demonstrate aberrant T-cell immunophenotype by flow cytometry or clonality by TCR gene rearrangement. Ultimately, these findings need to be considered within the context of clinical and morphologic findings in cases that are morphologically indeterminate for T-cell lymphoma. For particularly difficult cases, molecular characterization is being increasingly performed to aid in the diagnosis of mature T-cell lymphomas.

Conversely, bona fide lymphoma, particularly cHL, can also masquerade as acute EBV infection or CAEBV. Bahethi et al. report a case of cHL which was initially diagnosed as reactive lymphadenopathy and subsequently diagnosed as CAEBV, before ultimately being diagnosed as cHL-mixed cellularity subtype approximately six months after initial presentation [17]. Caution must be exercised when diagnosing EBV lymphadenitis if potential neoplastic diagnoses cannot be definitively excluded in the context of clinical and radiologic evidence suggestive of malignancy.

In summary, acute EBV infection should be considered in the differential diagnosis of typical and atypical lymph node and bone marrow histopathology, especially in adolescents and young adults. Review of the clinical history, ancillary laboratory tests, and peripheral blood smear is beneficial to gain additional clues to suggest a reactive state. Timely communication with the clinical team and request for EBV serology and/or PCR studies is key to avoiding a misdiagnosis of lymphoma. Methodical adherence to the steps listed in a checklist (Table 3) may serve as a guide to pathologists and help in focusing work up and avoiding diagnostic pitfalls.

**Table 3:** A recommended checklist to help guide the pathologic workup of a lymph node biopsy in children and young adults

1- Remember to include EBV infection in the differential diagnosis when dealing with typical and atypical changes of lymph nodes, especially in children and young adults.
2- Be familiar with morphologic changes and IHC staining patterns expected in EBV infection, especially in lymph node and bone marrow.
3- Always review and correlate with a concurrent peripheral blood smear whenever EBV infection is suspected.
4- Correlate with EBV serology and/or EBV viral load whenever EBV infection is suspected upon review of a lymph node biopsy or bone marrow.
5- Know lymphoproliferative disorders where EBER positivity is expected (and those where EBER positivity excludes the diagnosis, e.g., ALCL).
6- Learn all clinical syndromes, common and rare, associated with acute, subacute, and chronic EBV infection.
7- Compare and correlate with radiologic and clinical findings but be aware of the effect of patient triage and bias of specialization (e.g., a very sick patient with lymphadenopathy and fever may get a different work up if admitted to the infectious disease ward versus the hematology/oncology ward).

## Supplementary Information

**Additional file 1. **EBV-associated lymphoproliferative disordersincluded in the WHO Classification of Hematolymphoid Tumors, 5th edition,online beta version [1] .

## Data Availability

All slides/data are available as needed.

## Abbreviations

EBV: Epstein-Barr virus
HLH: Hemophagocytic lymphohistiocytosis
IHC: Immunohistochemical
EBER-ISH: EBV-encoded RNA in-situ hybridization
DLBLC: Diffuse large B-cell lymphoma
CAEBV: Chronic active EBV infection
NGS: Next-generation sequencing
cHL: Classic Hodgkin lymphoma
ALCL: Anaplastic large cell lymphoma
WHO: World Health Organization
AITL: Angioimmunoblastic T-cell lymphoma
TCR-G/B: T-cell receptor gamma/beta
HSTCL: Hepatosplenic T-cell lymphoma
