# Supplementary material for: Differentiating fulminant EBV infection complicated by HLH from Lymphoma: report of a case and a brief literature review
Source: Diagn Pathol. 2023 Feb 22;18:28. doi: 10.1186/s13000-023-01307-x (PMC9945358; doi:10.1186/s13000-023-01307-x)
Supplement: Supplementary file 1 — Additional file 1. EBV-associated lymphoproliferative disordersincluded in the WHO Classification of Hematolymphoid Tumors, 5th edition,online beta version [1] . [file 13000_2023_1307_MOESM1_ESM.docx]

Supplement: EBV-associated lymphoproliferative disorders included in the WHO Classification of Hematolymphoid Tumors, 5th edition, online beta version^1^

| **Mature B-cell neoplasms** |
| --- |
| Burkitt lymphoma^a^  *Large B-cell lymphomas*  EBV-positive diffuse large B-cell lymphoma (DLBCL)  DLBCL associated with chronic inflammation  Fibrin-associated large B-cell lymphoma  Lymphomatoid granulomatosis  Plasmablastic lymphoma^b^  *KSHV/HHV8-associated B-cell lymphoid proliferations and lymphomas*  Primary effusion lymphoma^c^  KSHV/HHV-8 positive germinotropic lymphoproliferative disorder^c^  *Lymphoid proliferations and lymphomas associated with immune deficiency and dysregulation*  Polymorphic lymphoproliferative disorders arising in immune deficiency / dysregulation  Lymphomas arising in immune deficiency / dysregulation  EBV-positive mucocutaneous ulcer |
| **Hodgkin lymphoma**  Classic Hodgkin lymphoma^d^ |
| **Mature T-cell and NK-cell neoplasms** |
| *Mature T-cell and NK-cell leukemias*  Aggressive NK-cell leukemia  Adult T-cell leukemia/lymphoma^e^  *Nodal T-follicular helper (TFH) cell lymphoma*  Nodal TFH cell lymphoma, angioimmunoblastic-type^e^  Nodal TFH cell lymphoma, follicular-type^e^  Peripheral T-cell lymphoma, NOS^e^  *EBV-positive NK-cell and T-cell lymphomas*  EBV-positive nodal T- and NK-cell lymphoma  Extranodal NK/T-cell lymphoma  *EBV-positive T-cell and NK-cell lymphoid proliferations and lymphomas of childhood*  Severe mosquito bite allergy  Hydroa vacciniforme lymphoproliferative disorder  Systemic chronic active EBV disease  Systemic EBV-positive T-cell lymphoma of childhood  **Stroma-derived neoplasms of lymphoid tissues**  EBV-positive inflammatory follicular dendritic cell sarcoma^f^ |
|  |

^a^Burkitt lymphoma (BL): WHO Classification of Hematolymphoid Tumors, 5th edition acknowledges the traditional subtypes of Burkitt lymphoma (endemic BL, sporadic BL, and immunodeficiency-associated BL) but recommends new subtypes (EBV-associated BL and EBV-negative BL).^1^

^b^Plasmablastic lymphoma is EBV-positive in approximately 60% of cases.^1^

^c^Primary effusion lymphoma and HHV8-positive germinotropic lymphoproliferative disorder are universally positive for HHV8 with frequent co-infection by EBV.^1^

^d^Classic Hodgkin lymphoma subtypes exhibit different frequencies of EBV positivity, with the highest rates of EBV positivity occurring in lymphocyte-depleted and mixed cellularity subtypes.^1^

^e^Adult T-cell leukemia/lymphoma; nodal TFH cell lymphoma, angioimmunoblastic-type and follicular-type; and peripheral T-cell lymphoma, NOS are EBV-negative T-cell lymphomas which often feature EBV-positive B-cells in the background.^1^

^f^EBV-positive inflammatory follicular dendritic cell sarcoma was previously termed “Inflammatory pseudotumor-like follicular/fibroblastic dendritic cell sarcoma.”^1^
